# 

**Published:** 1883-10

**Authors:** 


					﻿OCTOBER, 1883.
THIRTETH YEAR.
HALL'S
JOURNAL
OF
HEALTH
Guaranteed Circulation 200,000 Copies in 12* Months.
E. H. GIBBS, A.M., M.D., Editor,
No. 21 CLINTON PLACE, EIGHTH STREET NEW YORK.
TERMS: $1 a Year.
Single number, 10 cts.
				

